# Safety assessment of Acori Tatarinowii Rhizoma: acute and subacute oral toxicity

**DOI:** 10.3389/fphar.2024.1377876

**Published:** 2024-03-19

**Authors:** Jia Liu, Xin Ping, Shu-jie Sun, Jiali Yang, Ye Lu, Lin Pei

**Affiliations:** ^1^ School of Basic Medicine, Hebei University of Chinese Medicine, Shijiazhuang, China; ^2^ Central Laboratory, Hebei Academy of Chinese Medicine Sciences, Shijiazhuang, China; ^3^ Turbidity and Toxicity Laboratory, Hebei Key Laboratory of Turbidity, Shijiazhuang, China

**Keywords:** Acori Tatarinowii Rhizoma, acute toxicity, subacute toxicity, hematology, serum biochemistry, histopathology, general behavior

## Abstract

**Introduction:** Acori Tatarinowii Rhizoma (ATR) is a well-known traditional Chinese medicine that is used for treating neuropathic diseases. However, there is little information about the safety of ATR.

**Methods:** The present study evaluated the acute and subacute oral toxicity of a water extract of ATR in Institute of Cancer Research (ICR) mice. In acute trials, a single administration of extract at a dose 5,000 mg/kg body weight led to no clinical signs of toxicity or mortality, indicating that the lethal dose (LD50) exceeded 5,000 mg/kg. A subacute toxicity test was done using daily doses of 1,250, 2,500, and 5,000 mg/kg of the ATR extract for 28 days, which did not show any adverse clinical symptoms or mortality. However, the male renal organ index and urea level in mice given 5,000 mg/kg was obviously abnormal, which was consistent with pathological results and suggested that this dose might cause kidney injury.

**Results:** Doses of ATR lower than 2,500 mg/kg could be regarded as safe, although the potential cumulative effects of long-term use of high doses of ATR need to be considered.

**Discussion:** The study highlights the function of ATR in reducing blood lipids and provides a new idea for its widespread clinical use in the future.

## Graphical Abstract

**Figure:**
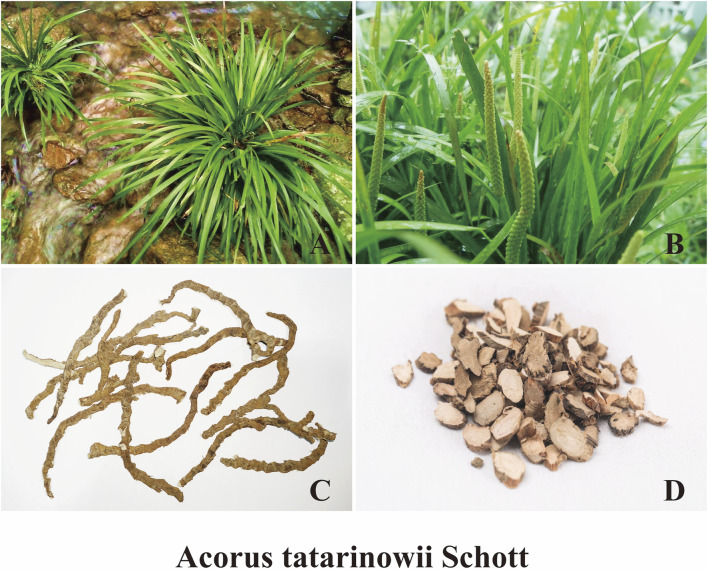
Representative image of Acori Tatarinowii Rhizoma. **(A)** Whole plant, **(B)** rhizomes, **(C)** dry rhizomes, **(D)** Chinese herb pieces.

## 1 Introduction

Acori Tatarinowii Rhizoma (ATR) is an important medicinal plant in traditional Chinese medicine, which is one of the most frequently used drug for treating brain diseases (Zhang et al., 2024), and over the past few decades, it has received continuous attention from medical and pharmaceutical researchers. According to reports, over 160 different structural types compounds have been identified in ATR. The involatile components include flavonoids, quinones, alkaloids, triterpenoid, saponins, phenylpropanoids, and organic acids, and the volatile components include α-asarone, β-asarone, methylisoeugenol, β-caryophyllene. Amino acids, sugars (Zhang et al., 2015), and trace elements have also been identified.

ATR has been shown to improve Alzheimer’s disease (AD) (Saki et al., 2020; Wang et al., 2021; Chen et al., 2022; Xiong et al., 2022) and shows outstanding performance as an antioxidant (Zhang et al., 2015) and anti-fatigue treatment (Zhu et al., 2014; Zhu et al., 2020). Significantly, ATR exhibits strong neuroprotective effects and reduces brain-nerve damage by improving cerebral blood circulation (Dong et al., 2014; Zhang et al., 2021). The key pharmacological components of Acori Tatarinowii Rhizoma extract are α-asarone and β-asarone. Modern pharmacological studies show that these active ingredients are widely used in the treatment of neurological diseases, such as depression, anxiety, epilepsy, and convulsions (Nandakumar et al., 2013; Koga et al., 2015; Liu et al., 2015; Tian et al., 2017; Zhang et al., 2019; Zhao et al., 2022; Xie et al., 2023), especially, α-asaronol promoted oligodendrocyte precursor cell differentiation and alleviated dysmyelination through the sensitization of PPARγ-GLT-1 signaling (Ge et al., 2022).

ATR is widely used as a natural medicinal plant and has broad development prospects, but related research has mainly focused on its clinical efficacy and pharmacological mechanisms (Li et al., 2020; Lu et al., 2023). There is little public safety information about its overall safety and side effects. The adverse reactions of natural medicinal plants have been attracting increasing attention in recent years (Traesel et al., 2016; You et al., 2020; Zhai et al., 2022). Therefore, in this study, a safety validation experiment was designed to explore the acute and subacute toxicity of ATR water extracts. The research provides a scientific basis for the clinical application of ATR and protecting public health and safety.

## 2 Materials and methods

### 2.1 Preparation of ATR water extract

ATR (batch number: 221001) was purchased from Anguo Yikang Pharmaceutical Co., Ltd. (Hebei, China) and satisfied the quality standards of the Chinese Pharmacopoeia (2020), as shown in graphical abstract. The sample was identified by Professor Xiao-gang Wang from the Pharmacy Department of Hebei Academy of Chinese Medicine Sciences (Hebei, China). ATR water extracts were prepared according to the following standard procedures.

A 1-kg sample of crude powder and 10 L of distilled water (ultra-pure water system, model OS007XXM1, ELGA, United Kingdom) were put into a multifunctional extraction tank (Shanghai Chaohong Instrument Equipment Co., Ltd., China), heated at 60°C, reflowed for 90 min, and filtered with 200-mesh screens. During the second extraction, 10 L of distilled water were added, extraction was done at 60°C for 60 min, and the extract filtered with 200-mesh screens. The extraction solution was concentrated to a relative density of 1.05–1.20 (60°C) using a rotary evaporator (Shanghai Yarong Biochemical Instrument Co., Ltd., China) at a vacuum of 80°C (pressure value 0.08 Mpa).

Next, the concentrate was spray-dried at 70°C, the spray powder was collected, and finally, a water extract powder of ATR was obtained. The brown powder was passed through a 50-mesh sieve and kept in a cool and dry environment (25°C, 35%–50% humidity) until use in experiments. ATR suspensions were freshly prepared daily with purified water before administration.

### 2.2 Experimental animals and housing conditions

Male and female Institute of Cancer Research (ICR) mice (6–8 weeks old and weighing 20–25 g) were provided by Beijing Vital River Laboratory Animal Technology Co., Ltd., Beijing, China, with Chinese animal use license SCXK (Beijing) 2021-0006. Before the experiment, the mice were allowed to adapt in cages for 7 days (22°C ± 2°C, 40%–70% humidity, light/dark reverse cycle for 12 h each). The mice were separated by sex and given free access to pure water and standard food. The animal experiments were conducted in accordance with the Good Laboratory Practice (GLP) principles of the National Medical Administration of China (NMPA) in the Animal Laboratory of Hebei Academy of Chinese Medicine Sciences and were approved by the Animal Care and Use Ethics Committee of Hebei University of Chinese Medicine (Ethics No. DWLL202302019).

### 2.3 Acute toxicity study

Acute oral toxicity studies were done according to the recommendations of protocol 423 of the OECD guidelines (OECD, 2001; Yang et al., 2019; Maimaiti et al., 2021; Ozan et al., 2023; Ru et al., 2023). The experiment was weighted and separated based on weight differences, and the 40 mice were divided into the following 4 groups of 10 each (5 males and 5 females). The groups received either no ATR water extract (0 mg/kg; control group), a low dose (1,250 mg/kg), a medium dose (2,500 mg/kg), or a high-dose (5,000 mg/kg). A volume of 20 mL/kg was administered by gavage.

At 16 h before the experiment, the animals were fasted. On the first day of administration, the mortality rate and general behavioural changes of all animals were regularly observed at 30 min, 1, 2, 4, 8, and 24 h. Starting from the second day, the mortality rate and abnormal clinical symptoms of the treated mice were observed every day for 14 consecutive days, as shown in Table 1. The median lethal dose (LD50) was determined using the acute toxicity method.

**TABLE 1:** Observation methods of toxicity experiment with ATR water extract in ICR mice.

Parameters	Content	Time
General objective	A. Changes in the colour of body surface hair, such as vertical hair, hair loss, and alopecia areata	Continuously for 1 h and intermittently for 4 h after each medication
	B. Skin rupture, redness, swelling, scars	
	C. Changes in body temperature, cool or hot	
	D. Tears or bloody tears, dilated or dilated pupils, protruding eyeballs, sagging or drooping upper eyelids	
Motor function	A. Spontaneous activity, combing of hair, changes in frequency of movement	
	B. Drowsy, prone to alertness	
	C. Dysfunction of movement, spasms and tremors, busyness, depression or immobility	
	D. Unconscious contraction of voluntary muscles	
	E. Convulsive contractions, clonic convulsions, syncope convulsions	
Respiratory system	A. Difficulty breathing, wheezing, and slowed breathing frequency	
	B. Shortness of breath or pause	
	C. Abdominal collapse during inhalation	
	D. Changes in nasal secretions	
Stimulus response	A. Changes in reflex ability to external stimuli (flipping, touching, noise)	
	B. Reduced responsiveness to pain stimuli or loss of pain sensation	
Secretions and excretions	A. Excessive secretion of saliva, vomiting or retching	
	B. Dry or watery stool	
	C. Haematuria, urinary incontinence	
Weight	Recorded changes in weight	Every day
Diet	Recorded consumption of food and water	
Organ index	Organ weight/body weight	End
Pathology	Dissection of all mice in the experiment. Recording of the position, colour, size changes of all organs and histopathological examination	
Blood	Blood routine and biochemical examination	
Death	Observation of the onset time, severity, and duration of poisoning symptoms. If the mouse dies, recording of the time of death and reaction and exploration of the reason	Every day

Body weight, food/water consumption, and corresponding changes were recorded daily. The mice received a known amount of food/water, and the remaining was measured 24 h later to determine the daily food/water intake as the difference. The mortality and abnormalities of the mice were also recorded. At the end of administration, mice were anesthetized with inhaled isoflurane, and then blood samples were taken. The animals were dissected, and organ weights were recorded.

### 2.4 Subacute toxicity study

Subacute toxicity experiments were performed for 28 days according to protocol 407 of the OECD guidelines (OECD, 2008; Yang et al., 2019; Maimaiti et al., 2021; Hisamuddin et al., 2023; Ozan et al., 2023; Ru et al., 2023). The experiments used 40 mice that were divided into 4 groups based on the same principle as the previous experiment with (10 mice in each group, 5 females and 5 males; control group (pure water), low-dose group (1,250 mg/kg), medium-dose group (2,500 mg/kg), and high-dose group (5,000 mg/kg)). All animals were gavaged at a weight of 20 mL/kg once a day for 28 consecutive days. The observations are shown in Table 1. Mice were anesthetized by inhalation using 4% isoflurane (batch number 20220803, Ruihua Life Technology Co., Ltd., Shenzhen, China) in the induction anesthesia room of the multi-channel small animal anesthesia device (R550, Ruihua Life Technology Co., Ltd., Shenzhen, China).

An anticoagulant vessel was used to collect about 0.5 mL of blood, which was immediately used in a fully automated blood cell analyser (Mindray, model BC6800 plus, Shenzhen, China). The following blood parameters were detected: white blood cell count (WBC), red blood cell count (RBC), haemoglobin (HGB), haematocrit (HCT), mean red blood cell volume (MCV), mean red blood cell haemoglobin (MCH), mean red blood cell haemoglobin concentration (MCHC), mean haemoglobin concentration (CHCM), red blood cell distribution width (RDW), red blood cell albumin distribution width (HDW), platelet count (PLT), mean platelet volume (MPV), neutrophils (NEU%), lymphocytes (LYM%), monocytes (MON%), eosinophils (EOS%), and basophils (BAS%). About 1.8 mL blood was collected and centrifuged at 3,000 rpm for 10 min to obtain serum for the detection of the following activity of the enzymes (Rayto Life Technologies, Chemray 800, Shenzhen, China): serum alanine aminotransferase (ALT), aspartate aminotransferase (AST), alkaline phosphatase (ALP), creatinine (CRE), total bilirubin (TBIL), glutamyltranspeptidase (GGT), urea, total protein (TP), albumin (ALB), glucose (GLU), total cholesterol (CHOL), triglycerides (TG), and the concentrations of sodium (Na^+^), chlorine (Cl^−^), and potassium (K^+^). The brains, hearts, livers, spleens, kidneys, lungs, testicles (males), uteruses and ovaries (females), thymuses, and bladders were weighed, and an organ index was calculated (absolute organ weight/body weight). All tissues and organs were stored in 10% neutral buffer formalin for histopathological examination.

### 2.5 Statistical analysis

The statistical analysis was conducted using the Windows version of GraphPad Prism 5.0 and plotted using Adobe Illustrator. The data were represented by the mean and standard deviation (SD). When the variance was homogeneous, the intergroup differences were evaluated using a one-way analysis of variance (ANOVA) and Dunnett’s test. When the variance was uneven, a Kruskal–Wallis nonparametric test was used. A difference was considered statistically significant at *p* < 0.05.

## 3 Results

### 3.1 α- and β-asarone quantification

**FIGURE 1:**
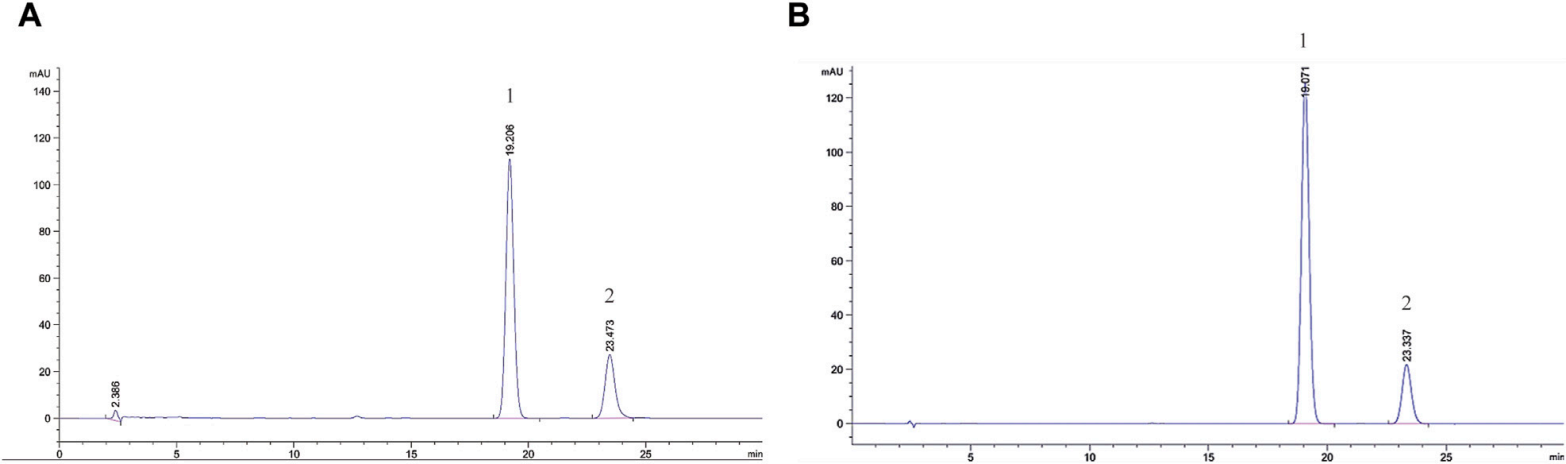
Comprehensive peak characterization trace showing Acori Tatarinowii Rhizoma and α- and β-asarone from HPLC analyses. **(A)** Sample map of water extract of Acori Tatarinowii Rhizoma, **(B)** Map of standard substance. 1: β-asarone. 2: α-asarone.

Figure 1 shows the chromatograms of α- and β-asarone quantification from 0.1 g of ATR extract and the peak maxima. The content of α- and β-asarone is approximately 6.63% and 14.68% in 0.1 g of ATR extract, respectively. The respective elution times of α- and β-asarone were recorded at 23.473 min and 19.206 min.

### 3.2 Acute oral toxicity in mice

#### 3.2.1 General behaviours

During the 14 days of observation, no animal deaths were observed (Table 2). Compared with the control group, there was no significant difference in weight and food/water consumption between each group (*p* > 0.05). The weight of the treatment groups was slightly lower than in the control group, as shown in Figure 1. All mice curled up for 1–5 min after gavage and then started normal activity. On the first day, two high-dose male mice showed less activity, while one medium-dose male mouse was insensitive to noise stimulation. On the third day, one low-dose and one high-dose female mouse had loose stool. These clinical signs all appeared briefly, and the mice recovered within 48 h, as shown in Table 2. The mice showed no other abnormalities.

**TABLE 2:** General behaviours in acute toxicity study with ATR water extract in mice.

Parameters	Male (n = 5)				Female (n = 5)			
	Control	Low-dose	Medium-dose	High-dose	Control	Low-dose	Medium-dose	High-dose
Appearance signs	0/5	0/5	0/5	0/5	0/5	0/5	0/5	0/5
Behavioral activities	0/5	0/5	0/5	2/5	0/5	0/5	0/5	0/5
Stimulus response	0/5	0/5	1/5	0/5	0/5	0/5	0/5	0/5
mental state	0/5	0/5	0/5	0/5	0/5	0/5	0/5	0/5
Secretions, excretions	0/5	0/5	0/5	0/5	0/5	1/5	0/5	1/5
Death	0/5	0/5	0/5	0/5	0/5	0/5	0/5	0/5

After dissection, there was no abdominal bleeding, and significant changes in organ colour and volume were observed. The organ coefficient values of the hearts, livers, spleens, lungs, kidneys, and brains were all within the normal ranges (Table 3). Therefore, we assumed that the LD50 of ATR exceeds the high dose based on these observations.

**TABLE 3:** Body weight, food/water consumption in acute toxicity study with ATR water extract in mice.

Parameters	Male (n = 5)				Female (n = 5)			
	Control	Low-dose	Medium-dose	High-dose	Control	Low-dose	Medium-dose	High-dose
Food intake (g/day)	5.34 ± 0.79	5.29 ± 0.63	5.18 ± 0.53	5.20 ± 0.53	5.21 ± 0.56	5.04 ± 0.55	5.09 ± 0.56	5.08 ± 0.59
Water intake (g/day)	5.28 ± 0.77	5.08 ± 1.08	5.16 ± 1.17	4.99 ± 1.49	6.33 ± 0.81	6.40 ± 0.77	6.33 ± 1.03	6.16 ± 1.17
Initial (g)	27.57 ± 0.69	27.26 ± 0.60	27.10 ± 0.56	27.13 ± 0.29	21.46 ± 0.91	21.40 ± 0.77	21.44 ± 0.45	21.18 ± 0.72
Final (g)	33.62 ± 0.60	32.78 ± 0.73	32.33 ± 0.80	32.30 ± 0.24	26.95 ± 0.84	26.43 ± 0.53	26.23 ± 0.81	25.95 ± 0.59
Change (g)	6.05 ± 0.58	5.52 ± 0.14	5.23 ± 0.64	5.17 ± 0.53	5.48 ± 0.64	5.03 ± 0.63	4.78 ± 0.84	4.77 ± 0.97

Values are expressed as the mean ± SD., No significant difference from control group (*p* > 0.05).

#### 3.2.2 Weight, consumption, post-mortem examination, and organ index

**FIGURE 2:**
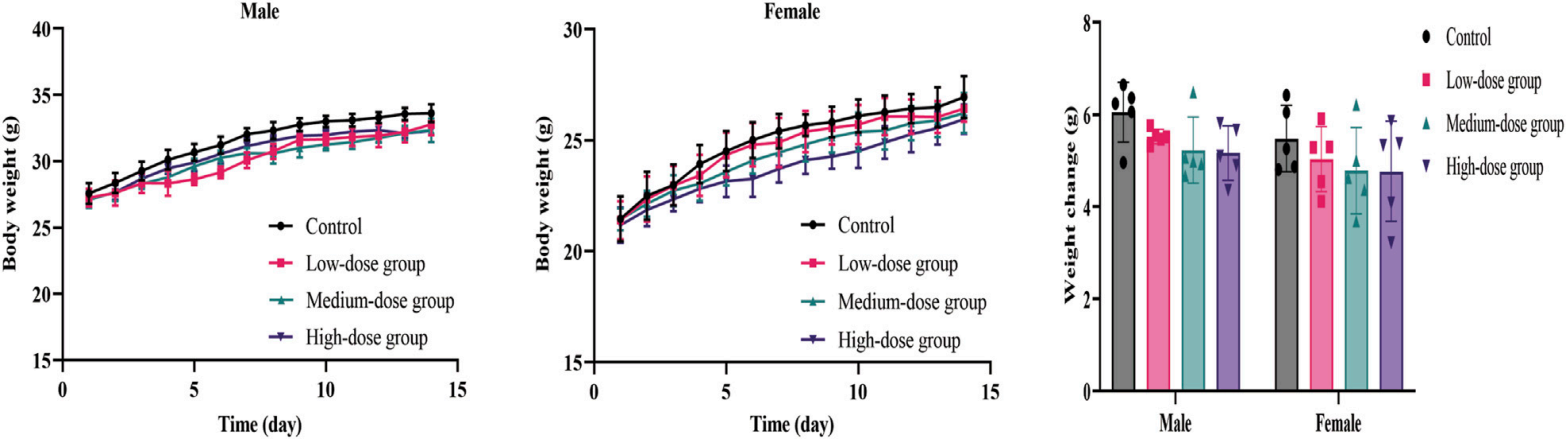
Mean body weight and weight changes of mice during the 14-day acute toxicity study. Error bars represent ±standard deviation.

The body weight and food/water consumption are shown in Figure 2 and Table 3. There were no significant changes in all groups (*p* > 0.05). The autopsy revealed no significant associated changes. There was no statistical significance for organ coefficients in each group (*p* > 0.05), as shown in Table 4.

**TABLE 4:** Organ index in acute toxicity study with ATR water extract in mice.

Parameters	Male (n = 5)				Female (n = 5)			
	Control	Low-dose	Medium-dose	High-dose	Control	Low-dose	Medium-dose	High-dose
Brain (g/100 g)	1.69 ± 0.071	1.72 ± 0.117	1.64 ± 0.081	1.82 ± 0.097	1.76 ± 0.056	1.69 ± 0.107	1.70 ± 0.128	1.83 ± 0.091
Heart (g/100 g)	0.54 ± 0.022	0.61 ± 0.071	0.55 ± 0.035	0.55 ± 0.048	0.53 ± 0.024	0.57 ± 0.024	0.57 ± 0.033	0.56 ± 0.034
Liver (g/100 g)	5.37 ± 0.288	5.32 ± 0.404	5.18 ± 0.450	5.55 ± 0.409	5.07 ± 0.403	5.21 ± 0.181	4.98 ± 0.286	4.77 ± 0.399
Spleen (g/100 g)	0.31 ± 0.024	0.33 ± 0.024	0.34 ± 0.032	0.34 ± 0.036	0.33 ± 0.019	0.36 ± 0.041	0.38 ± 0.056	0.38 ± 0.049
Lung (g/100 g)	0.51 ± 0.058	0.55 ± 0.091	0.59 ± 0.053	0.58 ± 0.058	0.64 ± 0.074	0.72 ± 0.041	0.71 ± 0.047	0.64 ± 0.058
Kidney (g/100 g)	1.62 ± 0.091	1.74 ± 0.076	1.65 ± 0.161	1.60 ± 0.077	1.43 ± 0.045	1.50 ± 0.057	1.35 ± 0.118	1.43 ± 0.039

All values represent the mean ± SD., No significant difference from control group (*p* > 0.05).

### 3.3 Subacute oral toxicity in mice

#### 3.3.1 General behaviours

There were no animal deaths or abnormal clinical symptoms associated with ATR. There were no obvious abnormalities in fur colour, behaviour, eating, drinking, breathing, or defecation in all mice. On the 10th day, depilation and dishevelled hair were observed in two low-dose male mice, but there was no alopecia areata, skin ulcers, or swelling. The condition was found to be related to a fight between male animals after observation. On day 17, slight wheezing and reduced activity were recorded in one medium-dose female mouse, which was possibly due to a manipulation error during gastric perfusion and returned to normal within 48 h. No other abnormal behavioural changes were seen in all groups, as shown in Table 5.

**TABLE 5:** General behaviours in subacute toxicity study with ATR water extract in mice.

Parameters	Male (n = 5)				Female (n = 5)			
	Control	Low-dose	Medium-dose	High-dose	Control	Low-dose	Medium-dose	High-dose
Appearance signs	0/5	2/5	0/5	0/5	0/5	0/5	0/5	0/5
Behavioral activities	0/5	0/5	0/5	0/5	0/5	0/5	1/5	0/5
Stimulus response	0/5	0/5	0/5	0/5	0/5	0/5	0/5	0/5
mental state	0/5	0/5	0/5	0/5	0/5	0/5	0/5	0/5
Secretions, excretions	0/5	0/5	0/5	0/5	0/5	0/5	0/5	0/5
Death	0/5	0/5	0/5	0/5	0/5	0/5	0/5	0/5

#### 3.3.2 Body weight and food/water consumption

**FIGURE 3:**
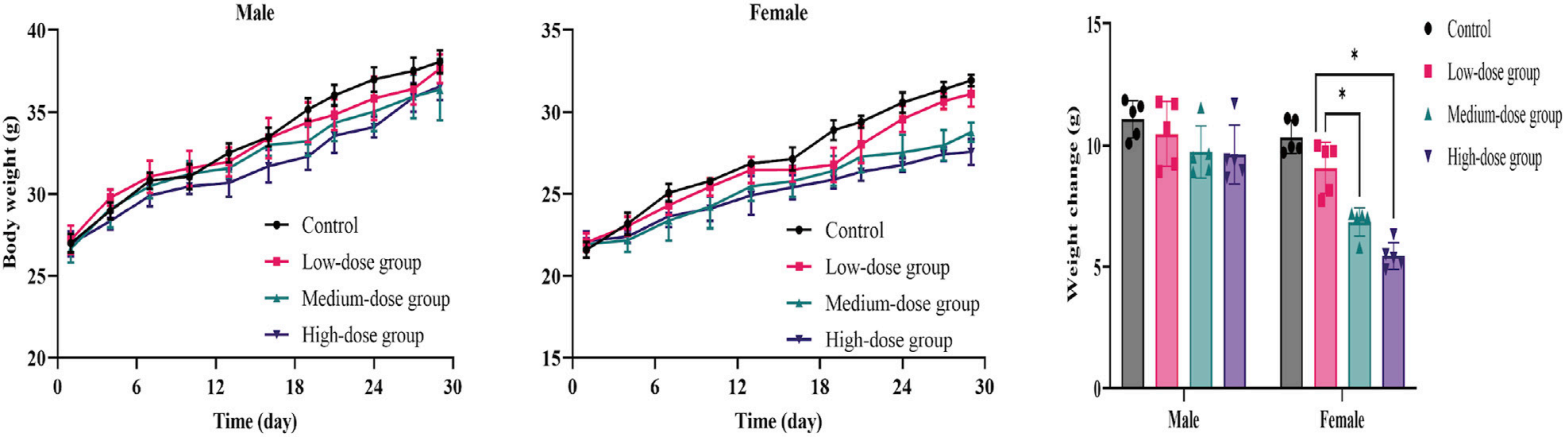
Mean body weight and weight changes of mice during the 28-day in subacute toxicity study. Error bars represent ±standard deviation.

The results showed that weight-grain rates of male mice treated with ATR slowed down. The weight-gain rates of male mice in the low-dose, medium-dose and high-dose groups were 38.5%, 36.5%, and 35.7%, respectively, which were lower than the control group (41.0%), but there was no significant difference. However, the body weight of female mice in medium-dose group and high-dose group was significantly different from that in the control group (*p* < 0.05). These mice showed increases of 31.2%% and 24.6%, respectively, which were lower than that in the control group (47.9%). There was no difference in the amount of food/water consumed by either group during administration. Therefore, ATR water extract did not damage the total food intake of the animals. The body weight and food/water intake are shown in Figure 3 and Table 6.

**TABLE 6:** Body weight, food/water consumption in subacute toxicity study with ATR water extract in mice.

Parameters	Male (n = 5)				Female (n = 5)			
	Control	Low-dose	Medium-dose	High-dose	Control	Low-dose	Medium-dose	High-dose
Food intake (g/day)	4.87 ± 0.59	4.75 ± 0.68	4.86 ± 0.73	4.74 ± 0.67	4.55 ± 0.63	4.44 ± 0.76	4.46 ± 0.63	4.41 ± 0.60
Water intake (m l/day)	4.10 ± 0.74	4.23 ± 0.62	4.26 ± 0.69	4.17 ± 1.06	5.09 ± 0.82	4.95 ± 0.78	4.98 ± 0.77	4.90 ± 1.10
Initial	26.99 ± 0.52	27.17 ± 0.78	26.64 ± 0.77	26.94 ± 0.67	21.58 ± 0.43	22.03 ± 0.50	21.92 ± 0.21	22.11 ± 0.55
Final	38.06 ± 0.64	37.64 ± 0.79	36.37 ± 1.68	36.56 ± 0.75	31.91 ± 0.32	31.10 ± 0.71	28.75 ± 0.53	27.55 ± 0.70
Change	11.07 ± 0.69	10.47 ± 1.20	9.72 ± 0.96	9.61 ± 1.08	10.33 ± 0.61	9.07 ± 0.94	6.83 ± 0.52*	5.44 ± 0.49*

Values are expressed as the mean ± SD. * Significant difference from the control group with *p* < 0.05.

#### 3.3.3 Post-mortem examination, organ index, and histopathology

After 28 days of administration, no significant abnormalities were found during the autopsy. The results showed that the thymus organ index was significantly higher in the medium-dose group and the high-dose groups of female mice than in the control group (*p* < 0.05), while no abnormality was observed in the male groups. However, this may have not been due to drug effects because there were abnormalities in the histopathological examination of thymus tissues in corresponding groups. Compared with the control group, the renal organ index of the male high-dose group had a statistically significant difference (*p* < 0.05), while the corresponding female mice had no change. The coefficient changes of other organs were not statistically significant (*p* > 0.05), as shown in Table 7.

**TABLE 7:** Organ index in subacute toxicity study with ATR water extract in mice.

Parameters	Male (n = 5)				Female (n = 5)			
	Control	Low-dose	Medium-dose	High-dose	Control	Low-dose	Medium-dose	High-dose
Brain (g/100 g)	1.60 ± 0.108	1.57 ± 0.037	1.58 ± 0.101	1.54 ± 0.052	1.64 ± 0.087	1.72 ± 0.094	1.76 ± 0.017	1.75 ± 0.120
Heart (g/100 g)	0.46 ± 0.035	0.49 ± 0.029	0.51 ± 0.040	0.51 ± 0.035	0.51 ± 0.026	0.55 ± 0.044	0.57 ± 0.034	0.57 ± 0.031
Liver (g/100 g)	5.00 ± 0.485	4.72 ± 0.418	4.70 ± 0.336	4.55 ± 0.298	5.13 ± 0.440	5.06 ± 0.319	5.01 ± 0.333	4.87 ± 0.379
Spleen (g/100 g)	0.37 ± 0.025	0.34 ± 0.029	0.33 ± 0.044	0.35 ± 0.047	0.34 ± 0.039	0.38 ± 0.054	0.39 ± 0.048	0.39 ± 0.076
Lung (g/100 g)	0.64 ± 0.021	0.61 ± 0.046	0.68 ± 0.040	0.66 ± 0.043	0.60 ± 0.068	0.54 ± 0.050	0.62 ± 0.077	0.68 ± 0.078
Kidney (g/100 g)	1.46 ± 0.109	1.53 ± 0.055	1.57 ± 0.101	1.72 ± 0.107*	1.33 ± 0.065	1.36 ± 0.135	1.39 ± 0.112	1.43 ± 0.069
Thymus (g/100 g)	0.16 ± 0.014	0.14 ± 0.019	0.16 ± 0.015	0.15 ± 0.012	0.17 ± 0.019	0.19 ± 0.016	0.23 ± 0.023*	0.27 ± 0.021*
Bladder (g/100 g)	0.06 ± 0.006	0.06 ± 0.008	0.06 ± 0.007	0.05 ± 0.006	0.06 ± 0.005	0.06 ± 0.008	0.07 ± 0.006	0.07 ± 0.006
Testis^a^ (g/100 g)	0.74 ± 0.051	0.70 ± 0.046	0.73 ± 0.075	0.72 ± 0.026	NA	NA	NA	NA
Ovary^b^ (g/100 g)	NA	NA	NA	NA	0.06 ± 0.005	0.06 ± 0.005	0.06 ± 0.005	0.05 ± 0.0005
Uterus^b^ (g/100 g)	NA	NA	NA	NA	0.31 ± 0.042	0.34 ± 0.062	0.28 ± 0.030	0.30 ± 0.045

Values are expressed as the mean ± SD. * Significant difference from the control group with *p* < 0.05.

^a^ Mean ± SD, for only male animals.

^b^ Mean ± SD, for only female animals.

**FIGURE 4:**
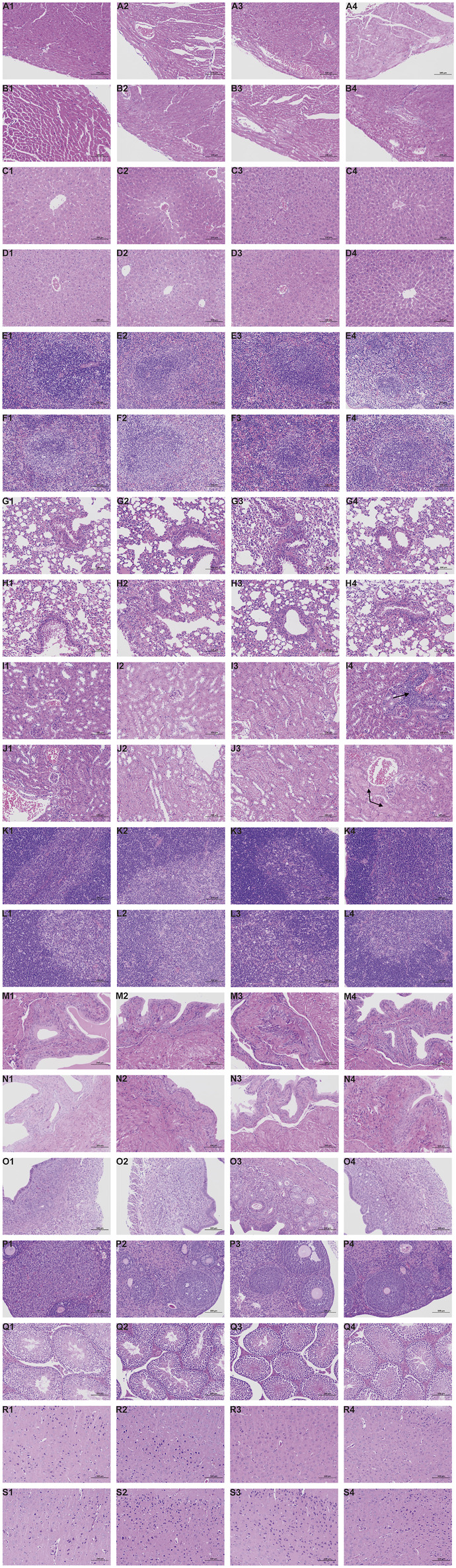
Histopathological results during the 28-day in subacute toxicity study. Stained with H&E, 200 ×, Scale Bars: 100 μm. Heart: A1 (Control, male); A2 (Low dose of ATR, male); A3 (Medium dose of ATR, male); A4 (High dose of ATR, male). Heart: B1 (Control, female); B2 (Low dose of ATR, female); B3 (Medium dose of ATR, female); B4 (High dose of ATR, female). Liver: C1 (Control, male); C2 (Low dose of ATR, male); C3 (Medium dose of ATR, male); C4 (High dose of ATR, male). Liver: D1 (Control, female); D2 (Low dose of ATR, female); D3 (Medium dose of ATR, female); D4 (High dose of ATR, female). Spleen: E1 (Control, male); E2 (Low dose of ATR, male); E3 (Medium dose of ATR, male); E4 (High dose of ATR, male). Spleen: F1 (Control, female); F2 (Low dose of ATR, female); F3 (Medium dose of ATR, female); F4 (High dose of ATR, female). Lung: G1 (Control, male); G2 (Low dose of ATR, male); G3 (Medium dose of ATR, male); G4 (High dose of ATR, male). Lung: H1 (Control, female); H2 (Low dose of ATR, female); H3 (Medium dose of ATR, female); H4 (High dose of ATR, female). Kidney: I1 (Control, male); I2 (Low dose of ATR, male); I3 (Medium dose of ATR, male); I4 (High dose of ATR, male). Kidney: J1 (Control, female); J2 (Low dose of ATR, female); J3 (Medium dose of ATR, female); J4 (High dose of ATR, female). Thymus: K1 (Control, male); K2 (Low dose of ATR, male); K3 (Medium dose of ATR, male); K4 (High dose of ATR, male). Thymus: L1 (Control, female); L2 (Low dose of ATR, female); L3 (Medium dose of ATR, female); L4 (High dose of ATR, female). Bladder: M1 (Control, male); M2 (Low dose of ATR, male); M3 (Medium dose of ATR, male); M4 (High dose of ATR, male). Bladder: N1 (Control, female); N2 (Low dose of ATR, female); N3 (Medium dose of ATR, female); N4 (High dose of ATR, female). Testis: Q1 (Control, male); Q2 (Low dose of ATR, male); Q3 (Medium dose of ATR, male); Q4 (High dose of ATR, male). Uterus: O1 (Control, female); O2 (Low dose of ATR, female); O3 (Medium dose of ATR, female); O4 (High dose of ATR, female). Ovary: P1 (Control, female); P2 (Low dose of ATR, female); P3 (Medium dose of ATR, female); P4 (High dose of ATR, female). Brain: R1 (Control, male); R2 (Low dose of ATR, male); R3 (Medium dose of ATR, male); R4 (High dose of ATR, male). Brain: S1 (Control, female); S2 (Low dose of ATR, female); S3 (Medium dose of ATR, female); S4 (High dose of ATR, female).

Representative pathological images of major organs in mice are shown in Figure 4. There were slightly thickened alveolar walls, a small increase in neutrophils, and occasional slight dilation of alveolar cavities in the lungs of each group, suggesting mild pneumonia. There was only mild cellular hydrodegeneration in the liver and kidneys, but there was no significant inflammation in each group and no significant difference between the administration groups and the control group. Therefore, we considered that the pathological change was spontaneous and not related to drug damage.

However, there was vacuolar degeneration of renal tubular epithelial cells and occasional minor glomerular capillary congestion in the female high-dose group. Similarly, in the male high-dose group, focal lymphocyte infiltration was observed in the renal cortex. In addition, compared with the control group, the renal organ index of the male high-dose group also showed significant differences, suggesting that the high dose may cause minor damage to the kidneys of mice.

#### 3.3.4 Hematology

The blood routine results of the mice from after the administration are shown in Table 8. Compared with the control group, the female low-dose group showed a significant decrease in HGB and MCH, although there was no dose-response relationship. Therefore, we assume that there was no toxicological significance. NEU (%) was significantly increased in both sexes of mice in the high-dose group compared to the control group, but the results were within the normal range for mice (52%–72%). PLT and MPV in the male group decreased with a dose-response relationship, but the difference was not statistically significant (*p* > 0.05). Therefore, we assume that the result had no toxicological significance.

**TABLE 8:** Haematological parameters of subacute toxicity study with ATR water extract in mice.

Parameters	Male (n = 5)				Female (n = 5)			
	Control	Low-dose	Medium-dose	High-dose	Control	Low-dose	Medium-dose	High-dose
RBC (10^12^/L)	10.15 ± 0.35	9.40 ± 0.88	10.39 ± 0.50	9.71 ± 1.28	10.30 ± 0.43	10.15 ± 0.35	10.04 ± 0.39	9.79 ± 0.63
HGB (g/dL)	145.06 ± 5.35	139.18 ± 6.93	146.78 ± 7.51	134.36 ± 11.57	149.06 ± 4.46	133.58 ± 7.10*	151.78 ± 8.88	137.98 ± 10.75
HCT (%)	42.94 ± 1.52	43.34 ± 1.35	42.38 ± 0.68	40.78 ± 1.70	43.18 ± 1.16	44.20 ± 2.10	42.56 ± 0.31	44.00 ± 1.10
MCV (fL)	43.22 ± 1.72	43.66 ± 2.21	42.78 ± 1.48	42.00 ± 1.26	44.86 ± 1.49	43.28 ± 1.19	43.34 ± 1.66	45.44 ± 2.85
MCH (pg)	14.04 ± 0.46	13.92 ± 0.53	14.48 ± 0.43	14.58 ± 0.62	14.56 ± 0.50	13.08 ± 0.62*	14.46 ± 0.27	14.34 ± 0.19
MCHC (g/dL)	294.80 ± 2.79	303.00 ± 12.66	288.00 ± 13.61	283.60 ± 12.42	293.00 ± 6.10	288.00 ± 3.16	298.00 ± 12.51	300.60 ± 4.22
CHCM(g/dL)	263.12 ± 3.91	260.34 ± 3.02	260.68 ± 5.45	258.50 ± 9.81	262.62 ± 4.08	262.12 ± 5.17	261.46 ± 6.02	256.46 ± 6.12
RDW(%)	16.00 ± 2.14	16.20 ± 1.02	15.04 ± 1.46	14.28 ± 1.67	15.36 ± 0.67	14.84 ± 0.40	15.68 ± 0.86	14.90 ± 0.96
HDW(g/dL)	21.36 ± 0.97	20.94 ± 0.30	22.08 ± 0.90	22.78 ± 1.39	21.02 ± 1.07	21.62 ± 1.10	21.80 ± 0.94	22.06 ± 0.52
PLT (10^9^/L)	1,100.20 ± 148.48	1,051.80 ± 69.64	1,020.00 ± 98.61	934.80 ± 115.72	1,111.80 ± 170.32	1,040.40 ± 160.20	1,036.20 ± 126.95	945.80 ± 168.47
MPV(fL)	7.44 ± 0.32	7.32 ± 0.31	7.24 ± 0.32	7.12 ± 0.89	8.46 ± 0.49	8.02 ± 0.77	8.00 ± 0.43	7.84 ± 0.41
WBC (10^9^/L)	7.55 ± 2.00	6.13 ± 1.77	8.35 ± 2.02	7.19 ± 2.50	7.20 ± 1.57	8.09 ± 2.17	6.63 ± 1.93	8.16 ± 2.22
NEU (%)	19.20 ± 3.78	18.36 ± 2.03	22.60 ± 5.35	34.72 ± 5.35*	19.00 ± 4.20	21.18 ± 2.96	24.50 ± 6.82	30.92 ± 2.44*
LYM (%)	67.60 ± 7.03	70.86 ± 7.06	65.20 ± 7.84	63.06 ± 7.68	70.64 ± 9.50	62.08 ± 4.78	57.62 ± 6.77	62.02 ± 6.93
MON (%)	1.94 ± 0.39	2.20 ± 0.23	1.72 ± 0.23	1.76 ± 0.39	1.88 ± 0.72	1.92 ± 0.27	2.14 ± 0.81	2.16 ± 0.84
EOS (%)	2.58 ± 0.70	2.20 ± 0.28	1.98 ± 0.50	1.82 ± 0.40	2.70 ± 0.84	2.48 ± 0.94	2.08 ± 0.67	1.94 ± 0.24
BAS (%)	0.72 ± 0.06	0.63 ± 0.09	0.65 ± 0.07	0.55 ± 0.15	0.63 ± 0.09	0.51 ± 0.10	0.60 ± 0.10	0.49 ± 0.23

Values are expressed as the mean ± SD. * Significant difference from the control group with *p* < 0.05.

#### 3.3.5 Serum biochemistry

The results of serum biochemistry are shown in Table 9. Compared with the control group, TG levels in female medium-dose and high-dose groups were significantly decreased (*p* < 0.05). The same happened in the male high-dose group (*p* < 0.05). Other data showed a downward trend in TC in the treated group of mice, although the result was not statistically significant. Furthermore, all values were within the normal range. Therefore, these changes were not considered to be adverse reactions associated with the test substance.

**TABLE 9:** Biochemical parameters of the subacute toxicity study with ATR water extract in mice.

Parameters	Male (n = 5)				Female (n = 5)			
	Control	Low-dose	Medium-dose	High-dose	Control	Low-dose	Medium-dose	High-dose
ALT (U/L)	37.52 ± 3.30	41.44 ± 5.52	43.86 ± 10.09	48.50 ± 11.96	33.84 ± 4.34	35.52 ± 3.69	42.64 ± 8.88	45.74 ± 12.91
AST (U/L)	135.26 ± 14.18	129.14 ± 17.39	145.96 ± 24.96	153.52 ± 33.28	128.64 ± 12.21	132.32 ± 7.50	139.42 ± 21.33	147.10 ± 22.18
ALP (U/L)	70.68 ± 9.40	76.80 ± 13.67	83.98 ± 10.88	87.92 ± 16.26	76.70 ± 6.31	81.04 ± 11.95	80.20 ± 8.29	90.20 ± 16.49
CRE (μmol/L)	33.94 ± 2.92	30.52 ± 6.10	28.28 ± 6.72	30.56 ± 7.60	29.28 ± 5.42	26.42 ± 2.28	26.68 ± 2.82	26.90 ± 4.39
TBIL (μmol/L)	2.87 ± 0.43	2.95 ± 0.63	3.10 ± 0.26	3.29 ± 1.26	2.78 ± 0.60	2.87 ± 0.68	3.01 ± 0.21	2.58 ± 0.70
GGT (U/L)	0.10 ± 0.01	0.09 ± 0.02	0.08 ± 0.02	0.08 ± 0.02	0.08 ± 0.02	0.10 ± 0.02	0.07 ± 0.02	0.07 ± 0.02
UREA (mmol/L)	7.27 ± 0.90	7.50 ± 0.72	7.65 ± 0.50	9.31 ± 0.56*	7.06 ± 0.57	7.12 ± 0.51	7.34 ± 0.42	7.27 ± 0.90
TP (g/L)	61.40 ± 2.76	60.10 ± 1.25	59.34 ± 3.40	58.62 ± 2.47	57.94 ± 2.77	56.66 ± 5.12	54.32 ± 4.22	53.58 ± 3.88
ALB (g/L)	31.48 ± 2.52	30.40 ± 2.48	28.08 ± 4.44	28.26 ± 2.65	26.10 ± 2.80	24.26 ± 2.99	23.88 ± 0.76	23.02 ± 1.89
GLU (mmol/L)	4.01 ± 0.38	3.73 ± 0.79	3.40 ± 0.64	3.31 ± 1.02	3.31 ± 0.32	2.91 ± 0.58	3.66 ± 0.64	4.06 ± 0.80
CHOL (mmol/L)	2.50 ± 0.59	2.18 ± 0.25	2.04 ± 0.48	1.89 ± 0.27	2.35 ± 0.36	2.29 ± 0.24	2.17 ± 0.12	1.95 ± 0.16
TG (mmol/L)	1.55 ± 0.31	1.46 ± 0.37	1.21 ± 0.31	0.92 ± 0.20*	1.46 ± 0.15	1.24 ± 0.28	1.16 ± 0.10*	1.01 ± 0.11*
K+ (mmol/L)	7.92 ± 0.41	7.54 ± 0.78	7.64 ± 0.77	7.02 ± 0.72	7.11 ± 0.46	6.78 ± 0.55	6.66 ± 0.50	6.40 ± 0.49
Na+ (mmol/L)	158.18 ± 2.30	159.82 ± 6.15	155.28 ± 3.62	155.22 ± 4.17	145.40 ± 2.80	149.28 ± 3.95	133.46 ± 5.67*	149.24 ± 3.58
Cl− (mmol/L)	97.00 ± 2.91	96.10 ± 2.01	99.40 ± 2.22	106.40 ± 1.77*	96.50 ± 5.44	100.22 ± 2.88	88.92 ± 4.89	98.22 ± 2.03
Ca2+ (mmol/L)	2.32 ± 0.15	2.10 ± 0.26	2.12 ± 0.31	1.98 ± 0.45	2.12 ± 0.17	2.08 ± 0.20	1.88 ± 0.21	1.82 ± 0.29

Values are expressed as the mean ± SD. * Significant difference from the control group with *p* < 0.05.

However, there was a statistically significant difference in urea between the male high-dose group and the control group, although the experimental values of the mice were within the normal range (Ozan et al., 2023). Compared to the control group, the Na^+^ concentration in the female medium-dose group was significantly decreased (*p* < 0.05), while the Cl^−^ concentration in the male high-dose group was decreased (*p* < 0.05). The experimental values of both were within the normal range for mice (Ru et al., 2023) and lacked a dose correlation. In addition, electrolytes in living organisms are dynamically changing and not static, so we considered this result to have no significant biological meaning and to be unrelated to toxicity. Other detection indexes were normal.

## 4 Discussion

The safety assessment of natural medicinal plants is a concern, especially for avoiding their adverse effects on health (Bernstein et al., 2021). The toxic potential of active ingredients present in natural medicinal plants has been extensively studied (Hudson et al., 2018; Roytman et al., 2018; Zárybnický et al., 2018). These studies involve safe doses and times, toxicological evaluations, and clinical application recommendations to reduce or avoid related adverse reactions. For hundreds of years, ATR has been used to treat diseases, but so far, evaluations of its toxicity have not been reported. Acute and subacute toxicity tests have been used to evaluate the toxicity or side effects of many natural extracts. Due to the lack of comprehensive information on its safety in the scientific literature, this study evaluated the safety of ATR extracts.

Mice are always one of the main mammals used in preclinical research, including pharmacological mechanism research and safety evaluations. Currently, international guidelines for chemicals and drugs require mice as models for safety assessment (Yang et al., 2019). Mice are small, low in cost, easy to obtain and study, and suitable for studying all stages of life, which makes them an ideal model organism for various experiments.

In the acute toxicity study, no deaths or adverse clinical symptoms were observed in each dose group, and no significant changes in body weight were observed. Therefore, according to the method of acute toxicity classification (Chushak et al., 2021), it can be considered that the plant extract is non-toxic. However, it is worth noting that long-term use of drugs may lead to accumulation in the body, gradually affecting tissues and organs. Therefore, as a continuation of acute toxicity studies, subacute toxicity tests were conducted using three doses of ATR extract.

Changes in weight in humans and animals directly reflect their overall health status, and there are many factors that affect it, such as genes, diet, and exercise. During the subacute toxicity experiment, although there was no difference in food intake among the groups, the weight of the female medium-dose group and female high-dose group decreased significantly. When acute poisoning occurs, animals may have symptoms such as drowsiness, anorexia, diarrhoea, and elevated liver enzyme levels (Amaral et al., 2022). In our study, no relevant symptoms were found, thus ruling out signs of toxicity.

Literature has shown that components of ATR raise HDL and lower triglyceride and total cholesterol levels, thereby lowering blood lipid levels, and it even showed comparable performance to simvastatin (Rodríguez-Páez et al., 2003; Argüelles et al., 2010; Mendieta et al., 2014; Thakare et al., 2016). In addition, other studies found am ethnic medicinal plant from the same genus, *Acorus calamus L.,* has therapeutic effects on diabetes and cardiovascular complications through a mechanism of insulin sensitization (Parab et al., 2002; Wu et al., 2009). Therefore, combined with the results of weight and biochemical tests which TG and TC showed a downward trend in this study, we speculated that ATR reduced blood lipids and changed metabolism, ultimately leading to weight loss, which was consistent with the results of previous studies (Lee et al., 2010). Significantly, the result is the same as the theory in traditional Chinese medicine.

The blood system is sensitive to toxic chemicals and is an important indicator for monitoring physiological changes in both humans and animals. Biochemical parameters are crucial for studying organs and tissues (Devi et al., 2022), especially in the kidneys and liver since one is used for excreting waste, and the other is used for uptake metabolism. In order to evaluate the toxicity of any new compound, it is necessary to understand the status of these two important organs, which can be validated through biochemical evaluation (Sureshkumar et al., 2018). Serum levels of three enzymes (ALT, AST, and ALP) are commonly used in studies as clinical biochemical markers related to liver injury (Yun et al., 2018). Our results showed no significant differences in their serum levels between all groups.

Histopathological examination provides a comprehensive assessment of the degree of injury and the specific organs affected. In addition, renal function can be evaluated by changes in urea, and changes in parameters indicate impaired renal function (Ezeja et al., 2014). The abnormal changes in ureal evels in our biochemical analysis support the pathological abnormalities in renal tissue at high doses. Accordingly, we assume that high-dose ATR extracts may have potential toxicity, which could lead to nephrotoxic side effects. However, the specific mechanism of the toxic effects and the chemical composition that may cause toxicity need further investigation.

However, the blood biochemistry results were not completely consistent with the pathological results. The pathological results showed that all groups had mild pneumonia, but there were no abnormal numbers of white blood cells, although they were generally high. This may have been due to the antibacterial and anti-inflammatory effects of ATR, which reduces white blood cells to some extent (Joshi, 2016). Therefore, in the pathological manifestations of pneumonia, the overall white blood cell count did not exceed the normal range. Blood tests are dynamic and variable results, unlike pathological results, which are long lasting and difficult to eliminate. In summary, the overall analysis of all ATR water-extract doses showed no significant damage to the important organs and reproductive organs of the treated animals, indicating that the low dose and medium dose are safe, while the high dose may cause minor kidney damage.

The no-observed-adverse-effect level (NOAEL) value was determined to be the medium-dose per body weight of ATR water extract. When converting the NOAEL of ATR water extract to the equivalent human dose (HED), it is calculated based on the body surface area and body weight (Nair et al., 2018). The HED value was calculated to be 191.65 mg/kg, which is equivalent to a dose of 11,499 mg for a 60-kg person, which is much higher than the clinical dosage for ATR prescribed by the Chinese Pharmacopoeia Committee (Committee of National Pharmacopoeia, 2020). These studies provide important information on the safety of oral ATR water extracts and support the sustained development and use of ATR water extracts as natural medicinal plants.

## 5 Conclusion

**FIGURE 5:**
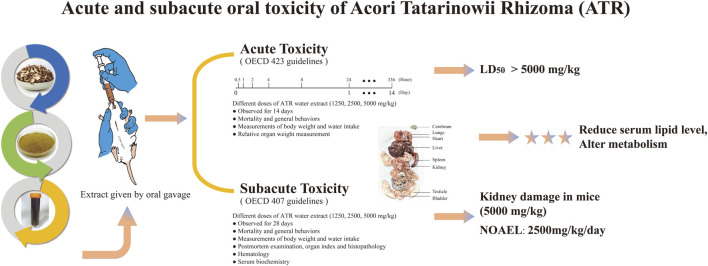
The schema graph of the experiment.

In conclusion, our research findings indicate that a single high-dose exposure to ATR water extract does not lead to acute toxicity in mice, but prolonged exposure to the high dose may lead to histopathological changes, especially in the kidneys. The results showed that the LD50 value of ATR water extract was greater than the high dose, and the NOAEL value was 2,500 mg/kg/day. However, in order to accurately determine NOAEL values, it is necessary to apply repeated subchronic doses for at least 90 days, as shown in Figure 5.

A surprising discovery was that ATR reduced serum lipid levels and altered metabolism. This could provide new ideas for the prevention of cardiovascular disease and the development of weight-loss drugs. As far as we know, this study is the first to report the safety assessment of ATR water extract and provides valuable preliminary data for the toxicity profile of ATR.

## Data Availability

The original contributions presented in the study are included in the article/Supplementary Materials, further inquiries can be directed to the corresponding authors.
